# Presence of a Novel Subtype of Bovine Hepacivirus in China and Expanded Classification of Bovine Hepacivirus Strains Worldwide into 7 Subtypes

**DOI:** 10.3390/v11090843

**Published:** 2019-09-11

**Authors:** Gang Lu, Jiajun Ou, Jiawei Zhao, Shoujun Li

**Affiliations:** 1 College of Veterinary Medicine, South China Agricultural University, Guangzhou 510642, China; LG@scau.edu.cn (G.L.); jj_inc@foxmail.com (J.O.); 18814113742@126.com (J.Z.); 2 Guangdong Provincial Key Laboratory of Prevention and Control for Severe Clinical Animal Diseases, Guangzhou 510630, China; 3 Guangdong Technological Engineering Research Center for Pet, Guangzhou 510630, China

**Keywords:** bovine hepacivirus, cattle, subtype, commercial bovine serum, China

## Abstract

The newest member of the *Hepacivirus* genus, bovine hepacivirus (BovHepV), was first identified in cattle in 2015 and is a novel hepacivirus C virus (HCV)-like virus. This virus has been detected in five countries so far and is classified into four subtypes. Bovine serum is commonly used for cell cultures and is considered the major source of viral contamination of pharmaceutical products. In this study, bovine serum samples were collected from seven countries located in Asia, America, Oceania, and Europe and were tested for BovHepV RNA using nested PCR, in order to: (i) obtain more knowledge on the geographical distribution and subtypes of BovHepV; and (ii) detect the potential contamination of BovHepV in commercial bovine serum samples used for cell culture propagation. The results demonstrated that bovine serum samples from individual donor cattle in China contained BovHepV RNA. After PCR, sequencing, and assembly, the genomes of the Chinese BovHepV strains were obtained. Genetic analysis of the polyprotein gene revealed a protein identity of <77% and a nucleotide identity of <85% between the Chinese BovHepV strains and all other previously reported BovHepV strains. Using cut-off values for determination of HCV genotypes and subtypes, BovHepV strains worldwide were classified into one unique genotype and seven subtypes. The BovHepV strains identified in the present study were classified into a novel subtype, which was provisionally designated subtype G. The genetic relationships among the different BovHepV subtypes were further confirmed through phylogenetic analysis. The present study provides critical insights into BovHepV’s geographical distribution and genetic variability.

## 1. Introduction

Hepatitis C virus (HCV) is one of the leading causes of hepatocellular carcinoma, cirrhosis, and liver failure in humans. Chronic hepatitis C virus (HCV) infection is estimated to affect >185 million persons worldwide [1]. Until recently, humans were considered the only established hosts for viruses in the *Hepacivirus* genus. Since 2011, HCV-like homologs have been discovered in several animal hosts, such as dogs [2], equines [3], bats [4], rodents [5], cattle [6,7], monkeys [8], shrews [9], sharks [10], turtles [11], and fish [11]. As suggested by the International Committee on Taxonomy of Viruses (ICTV), the *Hepacivirus* genus can be assigned to fourteen species (*Hepacivirus A–N*) and is classified within the *Flaviviridae* family, together with the genera *Flavivirus*, *Pegivirus,* and *Pestivirus* [12].

The genomes of viruses in the *Hepacivirus* genus are linear, positive-sense single-stranded RNA genomes ~10 kb in size with a single long open reading frame (ORF) flanked by a 5’ untranslated region (UTR) and a 3’ UTR. Each genome encodes a polyprotein that is further cleaved by host and viral proteases into three structural proteins (Core, E1, and E2) and seven nonstructural proteins (p7, NS2, NS3, NS4A, NS4B, NS5A, and NS5B) [6,7].

Bovine hepacivirus (BovHepV), a newly described hepacivirus that was found in the cattle population, is the only member of the *Hepacivirus N* species. This virus was identified for the first time in Ghana and Germany by two independent research groups in 2015 [6,7]. Several studies have indicated that BovHepV has a wide geographical distribution worldwide, including in Brazil [13,14], China [15,16], the USA [17], and Turkey [18]. Viral RNA screening has demonstrated that the prevalence of BovHepV in different countries ranges from 0.6% to 14.8%.

Similar to HCV, BovHepV is hepatotropic and can establish both acute and persistent infections in individuals [19]. Until now, 8 genotypes and 67 subtypes of HCV have been identified in humans [20]. According to a study by da Silva et al. in 2018, BovHepV is classified into one genotype (genotype 1) and four subtypes (subtypes A–D) based on the classification principle for HCV [14]. After this report, several investigations on the BovHepV genome in China, Turkey and Germany have been published, indicating that BovHepV is more genetically divergent than previously described [15,16,18,21].

In this study, we collected commercial bovine serum samples from seven countries in Asia, America, Oceania, and Europe and identified the presence of BovHepV RNA in these samples. We sequenced the near-complete genome sequences of three BovHepV strains, which were classified into a novel subtype. A total of seven subtypes of BovHepV were identified worldwide. This study indicates the importance of testing BovHepV in commercial bovine serum and increases our knowledge of the genetic diversity of BovHepV.

## 2. Materials and Methods

### 2.1. Sample Collection

A total of fifteen commercial bovine serum samples (sample ID: A–J) for cell culture were purchased from eleven serum manufacturers in seven countries located in Asia, America, Oceania, and Europe (Table 1). Three batches of each of serum J and K were used in this study, which were collected from individual donor cattle. Whether the other serum samples were pools or were collected from individual animals is unknown. Detailed information (i.e., age and gender) about these animals is unknown. After collection, these samples were immediately stored at −80 °C until further use.

### 2.2. Virus Detection

To detect BovHepV RNA in the serum samples, a total of 200 µL of each bovine serum sample was processed for RNA extraction using RNAiso Plus (Takara, China) according to the manufacturer’s protocol. The extracted nucleic acid was finally eluted in 20 µL of RNase-free water and was then used as the template for cDNA synthesis with random primers using a HiScript II 1st Strand cDNA Synthesis Kit (Vazyme, Nanjing, China). BovHepV RNA was tested by semi-nested PCR with primers targeting the conserved 5’ UTR. In this first round of PCR, the forward primer BovHepV3F (ATCRACACTCCAGGCTCAYG) and the reverse primer BovHepV264R (TGCGGCAGGACCCTATCA) were used. The following PCR cycling conditions were used: 35 cycles at 98 °C for 10 s, 55 °C for 15 s, and 72 °C for 30 s followed by 1 cycle at 72 °C for 5 min. In the second round of PCR, 1 μL of the first PCR product was used as the template, and another forward primer, BovHepV64F (AGTAGGAGGCGCCTATCCC), and the same reverse primer (BovHepV264R) were used. A bovine serum sample determined positive for BovHepV RNA in our previous study was used as a positive control [15], and PCR-grade water was used as a negative control.

After 1% agarose gel electrophoresis, a PCR product with the expected band size of 201 bp was considered BovHepV RNA positive, and the purified DNA was sent for direct Sanger sequencing from both ends (BGI, Shenzhen, China). A BLAST analysis was finally performed on the NCBI website [22] to determine whether the raw sequencing data contained BovHepV genomic information.

### 2.3. Viral Genome Sequencing and Analysis

All the BovHepV genomes available in the NCBI database were obtained and aligned by the ClustalW method using BioEdit 5.0.7.0. A total of five primer pairs covering the near complete genome of BovHepV were designed with Oligo 7.0 (Table 2). PCR was performed using Phanta Max Super-Fidelity DNA polymerase (Vazyme, Nanjing, China). After agarose gel electrophoresis and DNA purification, the amplified genome DNA fragments were cloned into a pCloneEZ-Blunt plasmid vector (CloneSmarter, Houston, TX, USA) and then transformed into *E. coli* DH5α competent cells (Weidi, Shanghai, China). The positive bacterial clones were sent for sequencing. The BovHepV genomes were assembled with SeqMan 7.1.0 based on the raw sequencing data. The nucleotide and amino acid identities of the polyprotein-coding sequences of the BovHepV strains were calculated using MegAlign 7.1.0.

**Figure 1:**
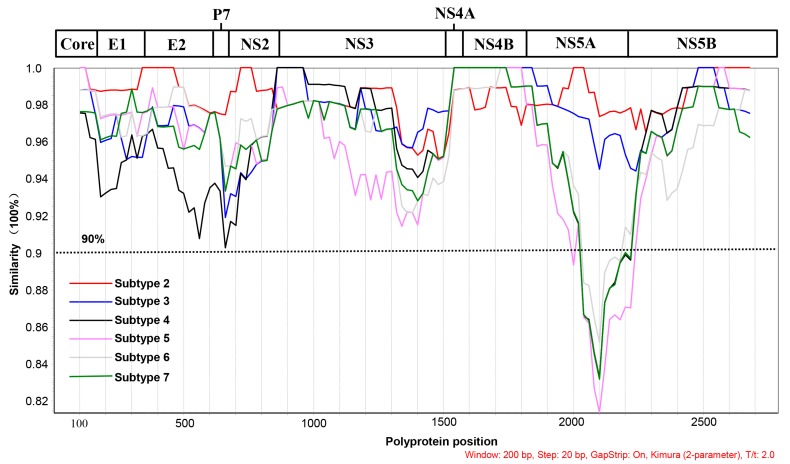
SimPlot analysis of BovHepV strains of subtypes A–G based on polyprotein. A schematic of the genome organization of BovHepV is shown above. The results of pairwise polyprotein comparisons of BovHepV subtype A against subtypes B–G are indicated. The cut-off value of 90% amino acid identity is shown by the dotted line. The strain names and accession numbers of the BovHepV strains of subtypes A–G are listed in Table 3.

In addition, a standard similarity plot analysis was performed using the polyprotein sequences of the BovHepV strains of subtype 1 as queries for comparison with other subtypes with the SimPlot v.3.5.1 software program under a window size of 200 bp and a step size of 20 bp (Figure 1).

To detect potential recombination events, BovHepV sequences were tested by seven methods (RDP, GENECONV, Chimaera, MaxChi, BootScan, SiScan and 3Seq) using Recombination Detection Program (RDP) version 4.27; an event with a *p* value of 0.01 and a recombination score of >0.6 was considered a possible recombination event.

### 2.4. Phylogenetic Analysis

**Figure 2:**
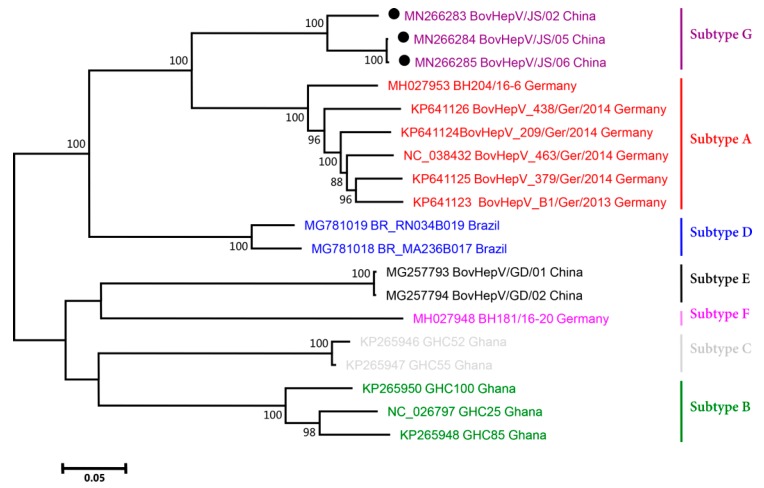
Phylogenetic analysis of BovHepV strains of subtypes A-G based on their polyprotein genes. The BovHepV strains identified in the present study (BovHepV/JS/02, BovHepV/JS/05, and BovHepV/JS/06) are indicated with circles. The detailed information for each BovHepV strain is shown, including the GenBank accession number, strain name, and country of origin.

To understand the genetic relationships between BovHepV strains, their polyprotein-coding sequences were aligned and then used to conduct phylogenetic analysis (Figure 2). After genetic distance was estimated with the “Find Best DNA Models” program, a maximum likelihood (ML) phylogenetic tree was established with the Tamura-Nei (TN93) and Gamma distributed with Invariant sites (G + I) substitution models using MEGA 5.05 with a bootstrap value of 1000 replicates.

## 3. Results

### 3.1. BovHepV Detection in Commercial Bovine Serum Samples

A total of fifteen commercial bovine serum samples from seven countries on four continents were collected to test for the presence of BovHepV RNA. After semi-nested PCR, sequencing, and BLAST analysis, three batches of serum K samples from one manufacturer in China were determined to be BovHepV RNA positive. According to the manufacturer, the three batches of serum samples were obtained from three independent donor cattle in Jiangsu Province, Central China (Table 1). All the other samples were BovHepV RNA negative. The three field BovHepV strains in China were designated BovHepV/JS/02, BovHepV/JS/05, and BovHepV/JS/06.

The PCR products targeted the 201 bp 5′ UTR of BovHepV. The sequenced 201 bp 5′ UTRs of BovHepV/JS/02, BovHepV/JS/05, and BovHepV/JS/06 had 100% nucleotide identity with each other and had 97.0–99.5% nucleotide identity with those of previously reported BovHepV strains.

### 3.2. Viral Genome Sequencing and Analysis

The nearly complete genomes of BovHepV/JS/02, BovHepV/JS/05, and BovHepV/JS/06 were obtained using PCR based on five primer pairs, sequenced, and assembled, and have been submitted to the GenBank database with the assigned numbers MN266283–MN266285.

The genomes of BovHepV/JS/02, BovHepV/JS/05, and BovHepV/JS/06 had the same genome organization as determined for other HCV-like viruses: 5′ UTR-core-E1-E2- p7-NS2-NS3-NS4A-NS4B-NS5A-NS5B-3′ UTR. The genomes of the three strains were 8679 nucleotides long, each including a 231-nucleotide-long partial 5′ UTR, an 8337-nucleotide-long polyprotein gene, and a 111-nucleotide-long partial 3′ UTR. No nucleotide inserts or deletions were observed among these three strains. The G + C contents of the genomes of BovHepV/JS/02, BovHepV/JS/05, and BovHepV/JS/06 were 52.0%, 51.3%, and 51.7%, respectively, which were equal to those determined in other strains available online (51.4–52.7%).

A novel genotype of HCV is classified if it has a sequence identity of <77% with other strains at the amino acid level; a novel subtype of HCV is classified if it has a sequence identity of <85% with other strains at the nucleotide level [12]. According to these criteria, BovHepV has previously been classified into one genotype and four subtypes [14]. The nucleotide and amino acid identities of the polyprotein-coding sequences of all the BovHepV strains available online, together with the three sequenced strains in the present study, were all calculated in this study (Table 3).

The results demonstrated that the amino acid identities of the BovHepV strains ranged from 91.7–100%, representing a unique genotype of BovHepV. The nucleotide identity among BovHepV/JS/02, BovHepV/JS/05, and BovHepV/JS/06 ranged from 92.7–99.9%. However, the three strains had a nucleotide identity of 79.2–84.5% with other BovHepV strains. These results indicated that they should be classified as a novel subtype, which was designated subtype G. In addition, the other BovHepV strains should be classified into six subtypes, i.e., subtypes A–F. Subtypes A and F included six and one German strain, respectively. Subtype D contained two Brazilian strains. Subtypes B and C included two and three Ghanaian strains, respectively. Two previously reported BovHepV strains (BovHepV/GD/01 and BovHepV/GD/02) in Guangdong Province in South China were classified into subtype E. The three sequenced field strains in the present study had a nucleotide identity of 79.2–79.8% with BovHepV/GD/01 and BovHepV/GD/02. The results clearly demonstrated that two subtypes of BovHepV were cocirculating in the Chinese cattle population.

To understand the changing trend of amino acid similarity in different polyprotein regions among the seven BovHepV subtypes, a SimPlot analysis was performed (Figure 1). When the polyprotein sequence of BovHepV subtype G was used as the query strain, the sequence of subtype G had a high amino acid similarity of >90% with the nearly complete polyprotein sequences of subtypes A–F, except for the NS5A protein, which had a low amino acid similarity of 81–90% with that of subtypes B, C, E, and F.

In addition, no potential recombination events within BovHepV strains were identified after systematic analyses were performed.

### 3.3. Phylogenetic Analysis

Phylogenetic analysis clearly classified the BovHepV strains into two main groups (Figure 2). One group included subtypes A, D, and G, and the other group included subtypes B, C, E, and F. The Chinese BovHepV strains of subtypes G and E had the closest genetic relationships with the German BovHepV strains of subtypes D and F, respectively. This finding was consistent with the results of the analysis of nucleotide similarity among different BovHepV subtypes shown in Table 3.

## 4. Discussion

Bovine serum is one of the most widely used biological reagents of animal origin for cell cultures and is considered the major source of viral contamination of pharmaceutical products. Bovine viral diarrhea virus (BVDV) and bovine parvovirus contamination have been identified in commercial fetal bovine serum [23]. Until now, investigations involving BovHepV isolation in vitro have not been published, and the tropism of BovHepV in cells derived from different species has not been determined. The present study confirmed the presence of BovHepV in commercial fetal bovine serum. Therefore, considering the potential of BovHepV to bias experimental results, it is necessary to test for BovHepV before using bovine serum for cell cultures.

With regard to newly discovered HCV-like viruses, genotyping and subtyping investigations have been restricted to only two viruses: equine hepacivirus (*Hepacivirus A*) and BovHepV (*Hepacivirus N*). Using cut-off values for HCV as references to determine genotypes and subtypes, equine hepacivirus has been classified into one genotype and three subtypes [24], and BovHepV has been classified into one genotype and four subtypes [14]. In this study, based on the same references, three other subtypes of BovHepV were identified, expanding the number of subtypes to seven. The genome sequences of only nineteen BovHepV strains (including the three newly sequenced strains in the present study) from four countries were available online and included in the subtyping investigations in this study (Figure 2). Cattle are widely distributed on all continents worldwide in very large numbers. Conducting an epidemiological survey on BovHepV worldwide and obtaining more BovHepV genomes may help us fully understand the BovHepV subtypes.

Notably, only one BovHepV strain (BH181/16-20) was classified into subtype F. This strain was reported by Schlottau et al. in an epidemiological investigation of BovHepV in Germany in 2018 [21]. The researchers sequenced the partial NS3 sequences of 31 German BovHepV strains. The sequences formed two independent clusters in phylogenetic analysis that contained 7 and 24 strains. Among the 31 strains, two BovHepV strains (BH204/16-6 and BH181/16-20) from each cluster were chosen for further sequencing of the complete polyprotein nucleotide sequence. Therefore, it is possible that >1 BovHepV strain in subtype F is circulating in the cattle population in Germany.

The present study determined the conserved nucleic acid and protein regions among different BovHepV subtypes. RNA-dependent RNA polymerase lacks a proofreading/repair function, and hepacivirus is considered highly genetically variable. In accord with this information, genomic analysis of the polyprotein gene indicated a nucleotide identity of 78.9–84.5% among different BovHepV subtypes (Table 3). However, after sequencing and alignment, it was observed that the nucleotide sequence of the 5′ UTR was conserved among the different BovHepV strains; thus, this sequence can be used as a potential target region in nucleic acid screening studies to detect all BovHepV subtypes. The SimPlot analysis of polyprotein demonstrated that the partial/entire NS3, NS4A, NS4B, and NS5B proteins had an amino acid identity of >95% among the different BovHepV subtypes (Figure 1). Currently, a serological detection method for BovHepV has not been systematically developed. Further study is still needed to understand the antigenicity and immunoreactivity of these conserved viral proteins and to establish a serological method to detect antibodies against all BovHepV subtypes.

Previous studies have demonstrated that HCV NS5A is a multifunctional protein that plays important roles in both viral genome replication and assembly [25]. NS5A is also a target for direct-acting antivirals in the treatment of HCV. In the present study, NS5A of BovHepV strains of subtypes B, C, E, and F was observed to be distant from NS5A of other subtypes at the amino acid level. Whether NS5A variation in BovHepV influences its replication and pathogenicity requires further investigation.

BovHepV RNA was only identified in three batches of serum K collected from one manufacturer in China. According to the manufacturer, serum K was treated with heat inactivation. In contrast, most of the samples from the other manufacturers were treated with a 0.1 µM filter. The different treatments might ultimately determine whether BovHepV RNA is present in commercial bovine serum samples.

In conclusion, BovHepV RNA was detected in commercial bovine serum for the first time. As bovine serum is commonly used for cell cultures, this study emphasizes the importance of testing for BovHepV RNA in commercial bovine serum. In addition, BovHepV strains worldwide were classified into seven subtypes, and a novel subtype of BovHepV was identified in the Chinese cattle population. Our study enhances existing knowledge regarding the genetic diversity of BovHepV.

## Figures and Tables

**Table 1:** The information on each bovine serum sample tested in the present study.

Serum Sample ID	Batch	Origin Country/Continent
A		Uruguay/America
B		France/Europe
C		New Zealand/Oceania
D		Brazil/America
E		Brazil/America
F		Uruguay/America
G		Israel/Asia
H		New Zealand/Oceania
I		Australia/Oceania
J	batch 1	China/Asia
	batch 2	
	batch 3	
K	batch 1	China/Asia
	batch 2	
	batch 3	

**Table 2:** The information on the PCR primers used for amplifying the BovHepV genomes in the present study. ^§^ Numbered according to BovHepV strain BovHepV_463/Ger/2014.

Primer Sequence (5′→3′)	Targeted Genomic Region ^§^	Product Length (bp)
TAGTAGGAGGCGCCTATCCC; TTNACCTTRTAYTCRCACCC	64–1583	1520
CTCCGTGGGTGCGAATACAA; TACAGCGYTGVACCATRGCR	1558–3394	1837
ACTTGGAYGTCTGCACTTGT; ACCAHGCCATACCRCTGTC	3200–4423	1204
GGGTGCTCCTGGRGTTTAC; CGAGCGATCCGACARTCRGT	4320–6321	2002
CCGCTCCRTGACTACGAGAC; CGAGACGGTATCCACAGCYC	6283–8739	2457

**Table 3:** The sequence identity within the BovHepV polyprotein gene at the nucleotide (upper right) and amino acid (lower left) levels. The strain names and accession numbers of the BovHepV strains of subtypes A–G are shown in different colors. The sequence identity was calculated using MegAlign 7.1.0.

	A						B			C		D		E		F	G		
	KP641123	KP641125	KP641127	KP641124	KP641126	MH027953	KP265950	KP265943	KP265948	KP265946	KP265947	MG781018	MG781019	MG257793	MG257794	MH027948	MN266283	MN266284	MN266285
KP641123 BovHepV/B1/2013		93.8	93.6	93.3	91.0	90.8	80.1	79.9	79.8	80.3	80.4	82.4	82.5	79.5	79.5	80.1	84.4	84.5	84.5
KP641125 BovHepV/379/2014	98.2		93.6	93.1	91.4	91.0	80.2	80.0	79.8	80.3	80.5	82.5	82.8	79.5	79.5	80.1	84.3	84.1	84.0
KP641127 BovHepV/463/2014	98.0	97.9		93.5	91.7	90.9	80.0	80.2	79.9	80.3	80.6	82.5	82.9	79.6	79.6	80.3	84.4	84.5	84.5
KP641124 BovHepV/209/2014	97.9	98.1	98.0		91.6	90.9	79.9	80.0	79.8	80.5	80.7	82.5	82.5	79.5	79.5	80.1	84.5	84.5	84.5
KP641126 BovHepV/438/2014	97.5	97.4	97.4	97.4		90.5	79.6	80.1	79.7	80.2	80.3	82.2	82.3	78.9	78.9	79.6	84.3	83.9	83.9
MH027953 BH204/16-6	97.3	97.2	97.6	97.3	97.1		79.5	79.8	79.7	79.8	80.1	82.3	82.5	79.5	79.5	79.6	84.0	83.9	83.8
KP265950 GHC100	92.9	93.3	93.0	93.1	92.7	93.0		90.9	90.4	82.7	82.8	80.7	80.5	82.0	82.0	81.6	80.2	80.0	79.9
KP265943 GHC25	93.0	93.1	93.1	93.0	92.8	93.0	98.6		92.0	82.7	82.9	80.4	80.7	81.7	81.7	81.4	80.3	80.0	79.9
KP265948 GHC85	93.1	93.4	93.2	93.3	92.8	93.1	98.7	99.0		82.4	82.7	80.2	80.5	81.7	81.7	81.1	80.0	79.8	79.8
KP265946 GHC52	92.3	92.5	92.3	92.4	92.3	92.0	95.5	95.6	95.5		98.4	80.9	80.9	81.5	81.4	81.3	79.8	79.9	79.9
KP265947 GHC55	93.0	93.2	93.1	93.1	92.9	92.7	96.2	96.3	96.3	98.8		81.1	81.1	81.6	81.6	81.5	80.0	80.1	80.1
MG781018 BR_MA236B017	93.8	94.4	93.9	94.1	93.9	94.3	93.5	93.1	93.3	92.8	93.4		93.9	80.2	80.2	80.0	82.2	82.1	82.1
MG781019 BR_RN034B019	93.6	94.2	93.7	93.9	93.8	94.0	93.5	93.2	93.4	92.5	93.2	98.1		80.7	80.7	80.0	82.4	82.4	82.4
MG257793 BovHepV/GD/01	92.1	92.2	92.1	92.1	91.7	92.0	95.4	95.3	95.2	94.4	94.8	92.7	92.6		99.8	81.0	79.8	79.3	79.3
MG257794 BovHepV/GD/02	92.1	92.3	92.2	92.1	91.7	92.1	95.4	95.3	95.3	94.5	94.9	92.8	92.6	100.0		81.0	79.8	79.2	79.2
MH027948 BH181/16-20	92.0	92.3	92.2	92.1	92.0	92.2	95.2	94.8	94.8	94.4	95.0	92.6	92.9	94.3	94.4		79.4	79.7	79.7
MN266283 BovHepV/JS/02	95.3	95.6	95.7	95.6	95.6	95.8	93.4	93.2	93.4	92.7	93.2	94.7	94.7	92.5	92.5	92.5		92.8	92.7
MN266284 BovHepV/JS/05	95.4	95.6	95.6	95.6	95.4	95.6	92.9	92.8	93.1	92.4	92.9	94.5	94.4	92.3	92.3	92.2	98.0		99.9
MN266285 BovHepV/JS/06	95.3	95.5	95.6	95.5	95.3	95.5	92.8	92.8	93.0	92.4	92.9	94.4	94.3	92.3	92.3	92.1	97.9	99.8	

## References

[1] Thomas D.L. (2013). Global control of hepatitis C: Where challenge meets opportunity. Nat. Med..

[2] Kapoor A., Simmonds P., Gerold G., Qaisar N., Jain K., Henriquez J.A., Firth C., Hirschberg D.L., Rice C.M., Shields S. (2011). Characterization of a canine homolog of hepatitis C virus. Proc. Natl. Acad. Sci. USA.

[3] Burbelo P.D., Dubovi E.J., Simmonds P., Medina J.L., Henriquez J.A., Mishra N., Wagner J., Tokarz R., Cullen J.M., Iadarola M.J. (2012). Serology-enabled discovery of genetically diverse hepaciviruses in a new host. J. Virol..

[4] Quan P.L., Firth C., Conte J.M., Williams S.H., Zambrana-Torrelio C.M., Anthony S.J., Ellison J.A., Gilbert A.T., Kuzmin I.V., Niezgoda M. (2013). Bats are a major natural reservoir for hepaciviruses and pegiviruses. Proc. Natl. Acad. Sci. USA.

[5] Drexler J.F., Corman V.M., Muller M.A., Lukashev A.N., Gmyl A., Coutard B., Adam A., Ritz D., Leijten L.M., van Riel D. (2013). Evidence for novel hepaciviruses in rodents. PLoS Pathog..

[6] Baechlein C., Fischer N., Grundhoff A., Alawi M., Indenbirken D., Postel A., Baron A.L., Offinger J., Becker K., Beineke A. (2015). Identification of a novel hepacivirus in domestic cattle from Germany. J. Virol..

[7] Corman V.M., Grundhoff A., Baechlein C., Fischer N., Gmyl A., Wollny R., Dei D., Ritz D., Binger T., Adankwah E. (2015). Highly divergent hepaciviruses from African cattle. J. Virol..

[8] Lauck M., Sibley S.D., Lara J., Purdy M.A., Khudyakov Y., Hyeroba D., Tumukunde A., Weny G., Switzer W.M., Chapman C.A. (2013). A novel hepacivirus with an unusually long and intrinsically disordered NS5A protein in a wild Old World primate. J. Virol..

[9] Guo H., Cai C., Wang B., Zhuo F., Jiang R., Wang N., Li B., Zhang W., Zhu Y., Fan Y. (2019). Novel hepacivirus in Asian house shrew, China. Sci. China Life Sci..

[10] Shi M., Lin X.D., Vasilakis N., Tian J.H., Li C.X., Chen L.J., Eastwood G., Diao X.N., Chen M.H., Chen X. (2016). Divergent viruses discovered in arthropods and vertebrates revise the evolutionary history of the Flaviviridae and related viruses. J. Virol..

[11] Shi M., Lin X.D., Chen X., Tian J.H., Chen L.J., Li K., Wang W., Eden J.S., Shen J.J., Liu L. (2018). The evolutionary history of vertebrate RNA viruses. Nature.

[12] Smith D.B., Becher P., Bukh J., Gould E.A., Meyers G., Monath T., Muerhoff A.S., Pletnev A., Rico-Hesse R., Stapleton J.T. (2016). Proposed update to the taxonomy of the genera *Hepacivirus* and *Pegivirus* within the *Flaviviridae* family. J. Gen. Virol..

[13] Canal C.W., Weber M.N., Cibulski S.P., Silva M.S., Puhl D.E., Stalder H., Peterhans E. (2017). A novel genetic group of bovine hepacivirus in archival serum samples from Brazilian cattle. Biomed. Res. Int..

[14] Da Silva M.S., Junqueira D.M., Baumbach L.F., Cibulski S.P., Mosena A.C.S., Weber M.N., Silveira S., de Moraes G.M., Maia R.D., Coimbra V.C.S. (2018). Comprehensive evolutionary and phylogenetic analysis of *Hepacivirus*
*N* (HNV). J. Gen. Virol..

[15] Lu G., Jia K., Ping X., Huang J., Luo A., Wu P., Li S. (2018). Novel bovine hepacivirus in dairy cattle, China. Emerg. Microbes Infect..

[16] Deng Y., Guan S.H., Wang S., Hao G., Rasmussen T.B. (2018). The detection and phylogenetic analysis of bovine hepacivirus in China. Biomed. Res. Int..

[17] Sadeghi M., Kapusinszky B., Yugo D.M., Phan T.G., Deng X., Kanevsky I., Opriessnig T., Woolums A.R., Hurley D.J., Meng X.J. (2017). Virome of US bovine calf serum. Biol. J. Int. Assoc. Biol. Stand..

[18] Yesilbag K., Baechlein C., Kadiroglu B., Baldan Toker E., Alpay G., Becher P. (2018). Presence of bovine hepacivirus in Turkish cattle. Vet. Microbiol..

[19] Baechlein C., Baron A.L., Meyer D., Gorriz-Martin L., Pfankuche V.M., Baumgartner W., Polywka S., Peine S., Fischer N., Rehage J. (2019). Further characterization of bovine hepacivirus: Antibody response, course of infection, and host tropism. Transbound. Emerg. Dis..

[20] Smith D.B., Bukh J., Kuiken C., Muerhoff A.S., Rice C.M., Stapleton J.T., Simmonds P. (2014). Expanded classification of hepatitis C virus into 7 genotypes and 67 subtypes: Updated criteria and genotype assignment web resource. Hepatology.

[21] Schlottau K., Wernike K., Forth L., Holsteg M., Hoper D., Beer M., Hoffmann B. (2018). Presence of two different bovine hepacivirus clusters in Germany. Transbound. Emerg. Dis..

[22] The NCBI Website. https://www.ncbi.nlm.nih.gov/.

[23] Toohey-Kurth K., Sibley S.D., Goldberg T.L. (2017). Metagenomic assessment of adventitious viruses in commercial bovine sera. Biol. J. Int. Assoc. Biol. Stand..

[24] Lu G., Ou J., Sun Y., Wu L., Xu H., Zhang G., Li S. (2019). Natural recombination of equine hepacivirus subtype 1 within the NS5A and NS5B genes. Virology.

[25] Shanmugam S., Nichols A.K., Saravanabalaji D., Welsch C., Yi M. (2018). HCV NS5A dimer interface residues regulate HCV replication by controlling its self-interaction, hyperphosphorylation, subcellular localization and interaction with cyclophilin A. PLoS Pathog..

